# Mechanism of Cationic Lipid Induced DNA Condensation: Lipid–DNA Coordination and Divalent Cation Charge Fluctuations

**DOI:** 10.1021/acs.biomac.4c00192

**Published:** 2024-07-16

**Authors:** Weiwei He, Serdal Kirmizialtin

**Affiliations:** † Chemistry Program, Science Division, New York University Abu Dhabi, Abu Dhabi, United Arab Emirates; ‡ Department of Chemistry, New York University, New York, New York 10003, United States

## Abstract

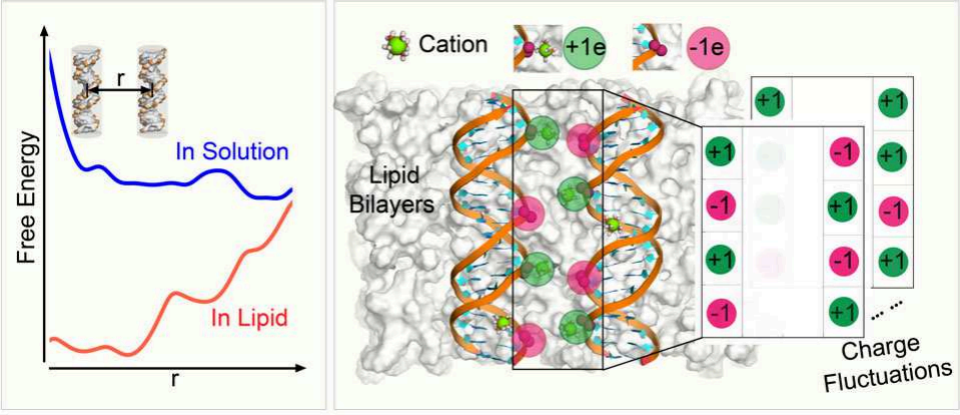

The condensation of nucleic acids by lipids is a widespread phenomenon in biology with crucial implications for drug delivery. However, the mechanisms of DNA assembly in lipid bilayers remain insufficiently understood due to challenges in measuring and assessing each component’s contribution in the lipid–DNA–cation system. This study uses all-atom molecular dynamics simulations to investigate DNA condensation in cationic lipid bilayers. Our exhaustive exploration of the thermodynamic factors reveals unique roles for phospholipid head groups and cations. We observed that bridging cations between lipid and DNA drastically reduce charges, while mobile magnesium cations “ping-ponging” between double strands create charge fluctuations. While the first factor stabilizes the DNA–lipid complex, the latter creates attractive forces to induce the spontaneous condensation of DNAs. This novel mechanism not only sheds light on the current data regarding cationic lipid-induced DNA condensation but also provides potential design strategies for creating efficient gene delivery vectors for drug delivery.

## Introduction

The condensation of DNA within confined spaces stands as a paramount factor in the intricate workings of cellular activities. Cell membranes, while serving as physical boundaries, also dynamically organize the assembly of biomolecules. This critical condensation process emerges as an indispensable element in the formation of membrane-bound organelles like mitochondria^1−4^ and chloroplasts.^5−8^ Condensation of DNA is also necessary for the efficient packaging of genomic DNA within the limited spatial constraints of the cell nucleus. The far-reaching implications of DNA condensation extend further, influencing not only the efficient packaging of genomic DNA within the limited confines of the cell nucleus but also governing the intricate phenomenon of chromatin phase separation. This phase separation, in turn, plays a pivotal role in shaping and organizing chromosomal subdomains within the nucleus.^9^ Thus, understanding the mechanism of DNA condensation in confinements is crucial to providing insights into the functional roles in cellular activities.^10−13^

Moreover, gene therapy has garnered significant attention as a promising technique for treating various diseases such as cancer, diabetes, and HIV/AIDS,^14,15^ as well as inherited genetic disorders.^16−18^ In this approach, nucleic acid (NA) drugs containing DNA or RNA chains are encapsulated and delivered to the interior of target cells while overcoming a range of biological defenses.^19,20^ The ability to tightly condense (package) nucleic acid cargoes within delivery vectors is a crucial prerequisite for the rational design of therapeutics in the realm of disease treatments.

Extensive research over the years has demonstrated that cationic lipids (CLs) can serve as gene delivery vehicles for the targeted delivery of extracellular DNA into the cell nuclei.^21−25^ CL-mediated DNA delivery relies, to a considerable extent, on the compacted state of the DNA duplexes, allowing the CL–DNA complexes to be of an appropriate size to pass through small openings in the cytomembrane.^24^ Positively charged liposomes have been widely used in applications for transfection of mammalian cells^26^ and also in the first COVID-19 vaccines authorized for human distribution,^27,28^ during which the aspect of NA condensation and assembly within CL systems has garnered considerable attention.^24,25,29^ DNA–anionic liposome complexes have also gained attention due to their low cytotoxicity.^30^

Most CL–DNA complexes adopt a lamellar phase structure (L_α_^c^) with DNA chains sandwiched between cationic lipids.^26,29,31,32^ The lamellar CL–DNA complexes comprise DNA monolayers arranged in one-dimensional (1D) structures with a defined separation between DNA molecules. These structures are situated between the bilayers of CL. It has been shown that when confined between CL membrane systems, such as a binary mixture of 1,2-dioleoyl-*sn*-glycerophosphatidylcholine (DOPC) and 2,3-dioleyloxypropyltrimethylammonium chloride (DOTAP) lipids,^31,33^ opposing DNA double strands form condensates in physiologically relevant solvent conditions.^29,31,34,35^ The binary mixtures of the saturated neutral and cationic lipids dimyristoylphosphatidylcholine (DMPC) and dimyristoyl trimethylammonium propane (DMTAP) can also induce condensation to form L_α_^c^ or L_β_^c^ phase structures in monovalent salt condition.^36,37^

How the repulsive nature of genomic DNA in an aqueous solution transforms into attraction within the confines of a cationic lipid (CL) membrane remains debatable.^29,35,38^ Previous efforts to understand the interaction of DNA with CL membranes are based on theoretical models and molecular simulations. Based on these studies, DNA condensation on CL membranes involves a different pathway compared to DNA condensation in three dimensions with multivalent ions.^29,38−40^ Nonlinear and linear Poisson–Boltzmann (PB) studies suggest a release of bound counterions as a mechanism of the entropic stabilization of DNA–membrane complexes,^41,42^ which has also been supported by experiments.^35,43−45^ The helicity of DNA charge distribution incorporated into theoretical models shows the attractive behavior between DNA molecules.^46^ Based on a linear Debye–Hückel treatment with helical DNA charge distribution and the assumption of low dielectric permittivity at the DNA core, divalent cation induced CL–DNA condensation has been proposed.^38,47^

In addition to theoretical models, computational studies have argued that the CL bilayers intercalated with DNA chains directly interact with DNA via phospholipid head groups. As a result of this interaction, a stable CL–DNA complex can be formed even in the absence of counterions.^48^ All-atom MD simulations by Morzy et al.^49^ showed that electrostatic (ES) interactions, through divalent cation bridging between lipid membranes and DNA, contribute to the stabilization of the membrane–DNA complex. Coarse-grained (CG) computer simulations^50−54^ suggest unique structural and mechanical properties for CL–DNA conjugates to explain the changes in the inter-DNA spacing with varying CL concentrations.^50^ For example, in explicit water dissipative particle dynamics simulations, Gao et al.^52^ proposed that the formation of CL–DNA complexes results in a notable release of DNA and lipid counterions, which could drive the DNA binding to the lipid bilayers.

In addition, the surface of the lipid is proposed to contribute to the charge compensation of the DNA. The reduction in electrostatic repulsion is observed with the change in the mole fraction of the positive head groups in the membrane mixture.^35,39,40,47^ Experiments also suggest that a row of divalent cations is positioned between DNA strands.^29^ However, the spatial arrangement of these interface cations and how they induce attraction between like-charge DNA pairs have not been thoroughly understood.

To test the hypothesis proposed and assess the governing factors leading to DNA like-charge attraction in lipid bilayers, we conduct a comprehensive computational investigation of a CL–DNA system with quasi-random genomic DNA chains intercalated into DOPC/DOTAP membrane bilayers in all-atom MD simulations. Explicitly mimicking a previous experimental study,^29^ our primary aim is to provide a direct comparison between experiment and simulation. Comparing the interhelical distances and the excess counterions probed by experiments, we assess the accuracy of MD simulations. Later, we elucidate the principles and mechanisms governing the self-assembly of double-stranded DNA pairs between lipid bilayers. To achieve our goals, we employ well-tempered metadynamics (WTMD) simulations that fully explore the conformational dynamics of DNA–DNA interactions in the presence of divalent Mg^2+^ salts and lipid bilayers. As a reference state, we also simulate the same DNA pairs in an aqueous solution of Mg^2+^.

Consistent with experiments, we find the same salt condition that leads to the repulsion of DNA pairs in the aqueous environment causes spontaneous condensation of DNA pairs in the presence of the CL bilayer. Our explicit-solvent, atomistic simulations paint a complex picture of how both lipids and confined cations contribute to DNA condensation in 2D. The data suggests that DNA charge compensation and surface charge fluctuations are the two major factors modulating the DNA–DNA interactions. Our analyses suggest that the positive head groups of both DOTAP and DOPC lipids in contact with the DNA backbone reduce the effective DNA charge. In particular, the negative phosphate head groups of DOPC act as electrostatic bridges, connecting the DNA backbone to the membrane via Mg^2+^ cations. The nearly neutralized DNA chains are further brought together through correlated motion of cation fluctuations. Drawing its parameters from molecular simulation, we formulated a statistical thermodynamics model that describes the attraction caused by cation fluctuations. Understanding the spatial distribution of interface ions and their role in inducing like-charge attraction has implications in various fields, including the design of colloidal formulations, the development of self-assembled materials, and the control of particle interactions in applications ranging from vaccine development to drug delivery.

## Methods

### Structural Modeling and Simulation Setup

We employed molecular dynamics (MD) simulations to investigate the condensation of DNA with a quasi-random sequence (GCA TCT GGGC TATA AAA GGG and its complement) within a membrane environment and compared its behaviors to that of free DNA in solution. All simulations were performed using the GROMACS 2018.5 software package.^55^ We generated the initial structure of the DNA duplex by Nucleic Acid Builder (NAB)^56^ and placed the two duplexes of the same sequence in the simulation box. Initial coordinates of membrane bilayers were constructed using the lipid bilayer builder from the CHARMM-GUI interface,^57^ resulting in a binary mixture of DOPC and DOTAP lipids (mole fraction of DOPC Φ_DOPC_ ∼ 0.6^29^), and the topological information was converted to the GROMACS^55^ format using the CHARMM-GUI force field converter.^57,58^

**Figure 1:**
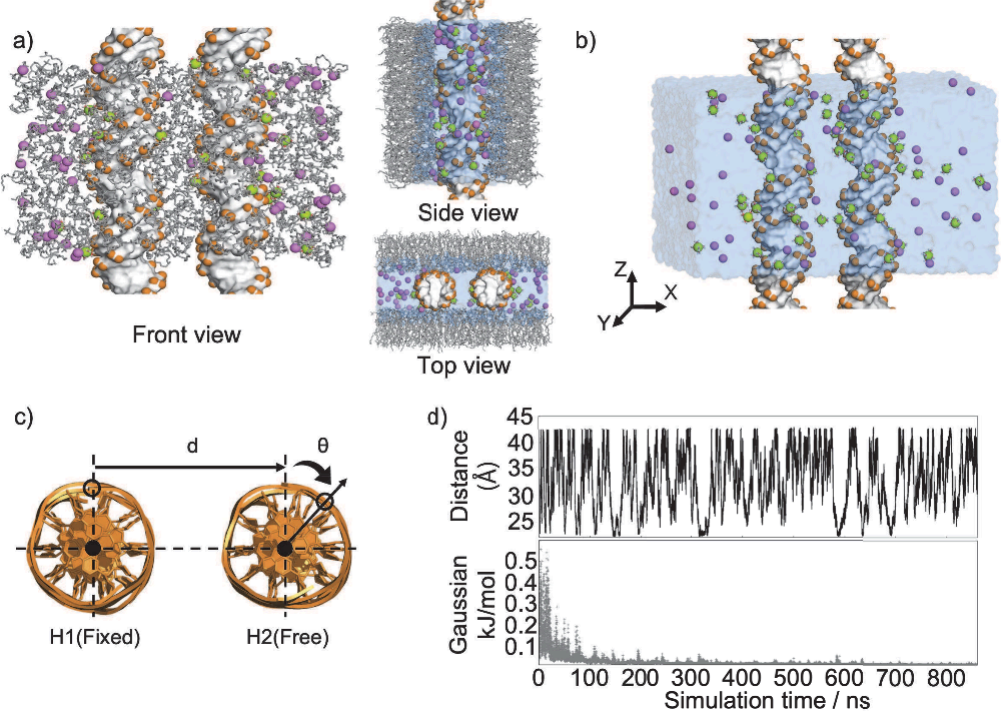
Simulation setup used to explore the energy landscape of inter-DNA conformational space. (a) DNA pairs were confined between cationic membrane bilayers, with purple beads representing Cl^–^ anions, green beads representing hexahydrated Mg^2+^ cations (Mg(H_2_O)_6_^2+^), and the lipid bilayers depicted as gray sticks. The periodic boundary conditions along the *z*-axis enabled the extension of the DNA system to infinite length, mimicking long DNA systems. (b) As a control, simulations of DNA in the absence of lipids were employed. Both setups used approximately 50 mM MgCl_2_ free cations to mimic experiments. (c) The two collective variables (*d*, θ) were used to navigate the energy landscape. To aid analysis, the orientation of helix 1 was restrained. The conformations were sampled using well-tempered metadynamics (WTMD) simulation (see Well-Tempered Metadynamics). (d) Fluctuations in the slowest degree of freedom, inter-DNA spacing *d* (top), and the convergence of time evolution of the deposited Gaussian heights (bottom) were used to assess the sampling efficiency.

During the simulations, we set the long axis of DNA constructs to align parallel to the box’s *z*-axis so that the opposing duplexes could be extended to infinite lengths due to periodic boundary conditions (Figure 1a,b).

We study the system in ∼50 mM free Mg^2+^ cations to mimic experiments.^29^ Full details of the simulation setup are provided in the Supporting Information (sections on simulation setup of DNA in solution and simulation setup of DNA in lipid membrane).

### Well-Tempered Metadynamics

Well-tempered metadynamics (WTMD) simulations were performed with the use of the PLUMED package.^59,60^ Details of the WTMD theory can be found elsewhere.^60^ As the biasing collective variables (CVs), we chose interhelical distance (*d*) and axial rotations of helix H2 (θ) (Figure 1c).^61^ We constrained the rotations and translations of helix H1 (Figure 1c) for ease of visualization and analysis. For the DNA-in-solution system, Gaussians with a height (*h*_G_) of 0.6 kJ·mol^–1^ and a sigma of 0.1 nm for *d* (σ_*d*_) and 0.2 rad for θ (σ_θ_) were deposited every picosecond. The bias factor γ was set to 8.0. For the DNA-in-lipid system, the Gaussian potential was deposited more gently (*h*_G_ = 0.2 kJ·mol^–1^ as the DNA pairs were confined to a limited space, σ_θ_ = 0.15 rad). Simulations were monitored for convergence of the free energy profiles (Figure S2); when the Gaussian heights deposited for the two CVs decayed below a set threshold (0.01 kJ·mol^–1^) we assumed the free energy surface (FES) was covered. In addition, block analysis was used to evaluate the convergence of simulations (Figure S1).

### Computing Scattering from MD Trajectory

To obtain the scattering profile from the instantaneous atom positions, we compute the electron density of the atoms in real space ***R***, *A*(***R***), as
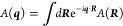
1where *A*(***q***) is the Fourier transform of the electron density of the coordinates and ***q*** is the scattering wave vector. The magnitude of ***q*** is determined by the scattering angle 2θ according to *q* = (4π/λ) sin θ, with the wavelength of the X-ray beam, λ.

The scattering intensity is then computed by
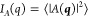
2Here, ⟨.⟩ represents the average over conformational states, keeping the translational and rotational degrees of the simulation system fixed. We used the WAXSiS program implemented in GROMACS-SWAXS to compute the scattering intensities.^62^

### Concentration Profiles

To study the distribution of cations and phosphate backbone, we projected particles along the interhelical distance. To do this, we first pick the starting points (*d* = 25 Å, 30 Å, 35 Å, 40 Å) from metadynamics trajectories and generate a pool of structures by sampling each state through 200 ns-long MD simulations (see Figures S4 and S5 for the lipid fluctuations and Figure S6 for the cation fluctuations), with interhelical distances *d* restrained to the defined states. We then compute the concentration profiles along the *x*-axis from the MD trajectory as
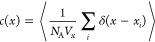
3where *V*_*x*_ is the solvent accessible volume between *x* and *x* + d*x*, *N*_A_ is Avogadro’s number, δ(*x*) is Dirac’s function, and ⟨.⟩ is the time average.

## Results and Discussion

By exhaustively sampling the conformational space of inter-DNA spacing and orientations (see Figure 1), we obtain thermodynamically converged ensembles of DNA–DNA interactions. We explore how the energy landscape of DNA–DNA interactions is influenced by the presence of the CL lipid bilayer. Additionally, we investigate the factors favoring DNA condensation within the lipid bilayer while also studying DNA pairs in aqueous solution for comparison. By carefully chosen potentials for ion–DNA and ion–ion interactions, we delve into the extent of counterion partitioning on both DNA and membrane surfaces. The contrasts in free energy profiles and solvent environments between these two scenarios enable us to uncover cation-mediated like-charge attraction of DNA double strands occurring within lipid bilayer membranes.

### Lipid Bilayers Induce DNA Condensation

**Figure 2:**
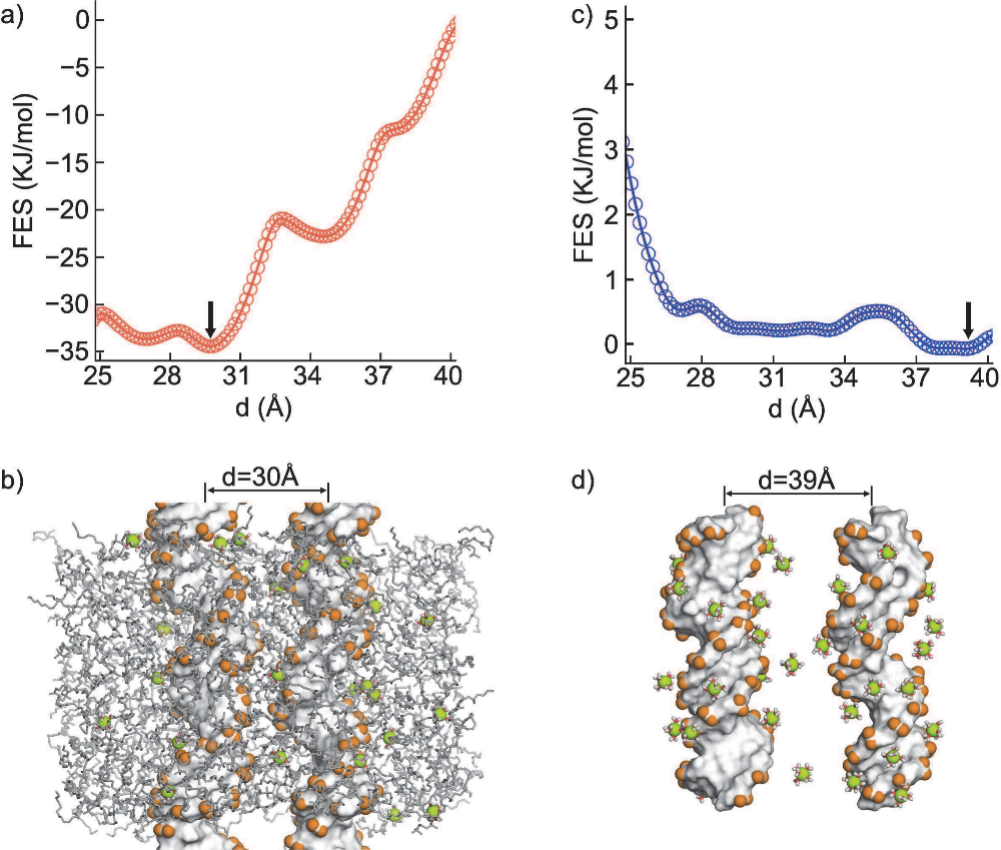
Free energy profiles of DNA–DNA interactions projected along the interhelical distance. (a) Free energy profile of DNA in the lipid bilayer. (b) A representative snapshot at its energy minimum. (c) Free energy profile of same pairs in solution. (d) A representative conformation at its minimum energy state.

Synchrotron X-ray diffraction experiments reported that divalent electrolyte counterions can condense anionic DNA molecules (linear λ-phage DNA) confined to the surfaces of cationic membranes.^29^ To elucidate the thermodynamic factors governing condensation, we employed WTMD simulations to construct the free energy landscape of DNA assembly in the presence of a lipid bilayer. To further validate the accuracy of the force field and investigate the free energy profile without the membrane, we examined the same DNA sequence in the absence of lipids retaining similar Mg^2+^ salt conditions. The free energy landscapes of the two setups are shown in Figure 2, where we projected the two dimensions onto the interhelical distance, *d*, for ease of analysis. Parts a and c of Figure 2 depict the variations in free energy as a function of interhelical distance, while parts b and d of Figure 2 illustrate the lowest energy states.

In the presence of membranes, we observed attractive DNA–DNA interactions, characterized by a prominent global minimum at approximately *d* ∼ 2.97 nm. This result is consistent with previous experimental studies reporting an interaxial distance of *d* = 2.89 ± 0.05 nm.^29^ Additionally, we identified two additional local minima at distances of 2.7 and 3.5 nm, likely arising from the discrete binding of DNAs on the membrane surface.

The presence of deep minima at close inter-DNA distances in lipid–DNA systems suggests spontaneous condensation, supporting experimental findings.^29^ However, in the free energy profile of DNA in solution, we observed a monotonic increase in the energy as the interhelical distance decreased (Figure 2c), contrasting with the profile observed in the presence of the membrane. The increasing energy as the DNA duplexes approached each other indicates repulsion between the DNA pairs. This finding is consistent with experiments, as only multivalent cations with a valence of *Z* = 3 or higher, such as biological polyamines and inorganic cobalt hexamine (Co(NH_3_)_6_^3+^), are known to induce attraction between genomic DNA duplexes.^21,63−65^

### Computed X-ray Diffraction Profiles Show Experimentally Consistent Assembly Process

**Figure 3:**
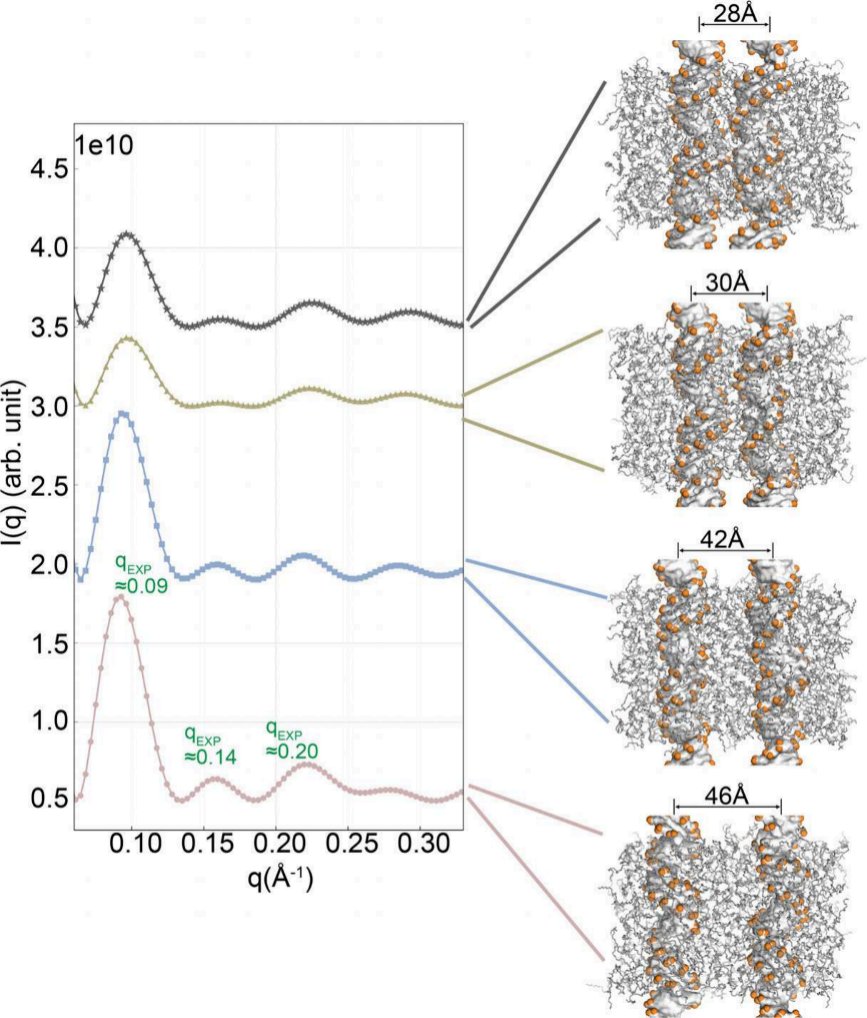
Computed X-ray scattering profiles. Computed X-ray scattering from lipids, DNA pairs, and ions at different interhelical distances aims to relate changes in the scattering profile with changes in real space, thereby establishing a connection between our findings and experimental observations.^29^ The peak positions corresponding to the experimental profile^29^ measured at *d* ≈ 47 Å are highlighted in green. Snapshots from simulations corresponding to each interhelical distance are shown on the right.

The equilibrium interhelical distance computed from the free energy profile (Figure 2) shows excellent agreement with the interhelical distance measured (2.97 nm vs 2.89 nm, respectively), which served as a benchmark for the study. To verify the consistency of the conformational states of the membrane–DNA complex sampled in simulations with experimental data, we computed X-ray diffraction profiles of the DNA–membrane system in explicit water and ions, as described in Computing Scattering from MD Trajectory. Using conformations from MD simulations at specific interhelical distances, *d*, we calculated the scattering curves and directly compared them with experimental data.^29^ Our analysis focused on the scattering features up to *q* = 0.3 Å^–1^ (Figure 3).

The obtained profiles illustrate the sensitivity of the scattering data to the positioning of DNA pairs, providing further evidence that the observed differences in scattering profiles between theoretical and experimental methods (Figure 2 in ref (29) and Figure 3) stem from the spatial positioning of DNA. We acknowledge that the some of the peaks are shifted and amplitudes in simulations were lower compared to the sharp peaks observed in experiments. This discrepancy in amplitudes can be attributed to the finite size of the simulation system, as experiments typically measure a larger base pair region compared to the computational methods that focus on a DNA pair of 20 base pairs. Nonetheless, the profiles exhibited remarkable similarities in terms of their overall features. The peak intensity at *q* ∼ 0.1 Å^–1^ indicates the overall size of the complex and shows a decrease as the DNA pairs approach each other. The periodicity of *d* = 2π/*q* ∼ 67 Å is equal to the combined thickness of the lipid bilayer and DNA monolayer in the experiment as well as our simulations. Similarly, the third peak reports the alternating structure of the lipid bilayer and DNA monolayer.^29^ We observed a shift of the second peak to higher values as the DNA pairs approach each other, in parallel with the reduction in the inter-DNA spacing.

### Cation Distributions Show Dramatic Differences between the Two Systems

**Figure 4:**
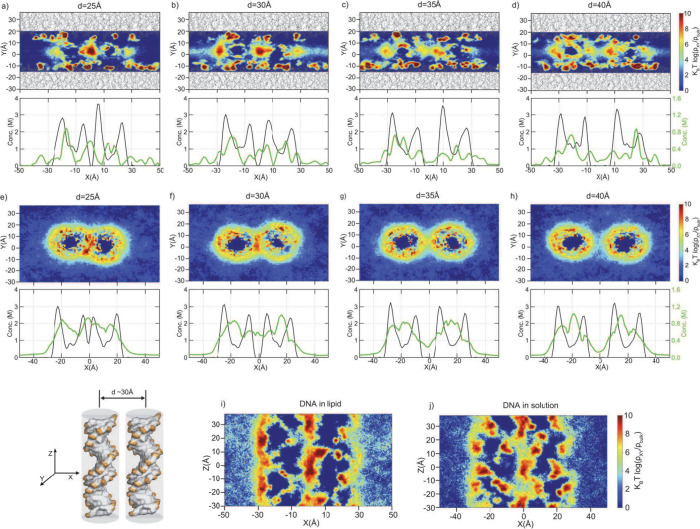
Average distribution of cations as a function of interhelical distance. (a–d) The average concentrations of Mg^2+^ at different interspacings computed for DNA pairs in lipid bilayers (depicted by gray lines). (top) Densities of Mg^2+^ ions projected onto the *x*–*y* plane. (bottom) The average densities of Mg^2+^ ions (green) and DNA phosphate (black) projected onto the *x*-axis. (e–h) Same as in (a)–(d), but this time for DNA pairs in solution. (i, j) Density profiles of Mg^2+^ on the cross section at *d* ∼ 30 Å for DNA (i) in a lipid environment and (j) in a solution environment.

The agreement observed between the simulations and experiments regarding the thermodynamics of the condensation process probed by the free energy calculations provided strong motivation to investigate the molecular details underlying the distinct behavior of DNA in lipid environments. The equilibrium interhelical distance computed from the free energy profile (Figure 2) shows good agreement with the interhelical distance measured (2.97 nm vs 2.89 nm, respectively), which served as a benchmark for our study. To assess if the simulation and experimental setup are similar, we computed SAXS profiles from all atom simulations. The agreement between simulation and experiment on the overall profile suggests our model is mimicking the experimental setup. We then computed SAXS profiles along the lowest energy points at varying interhelical distances. The semiqualitative comparison accomplished by comparing SAXS profiles at various salt conditions and computed SAXS profiles at various interhelical distances further verified that the second peak corresponds to the interhelical distance. To investigate the condensation induced by the lipid bilayer, we examined the distributions of Mg^2+^ ions. We performed a series of independent molecular dynamics (MD) simulations, constraining the inter-DNA distances to intermediate values along the assembly pathway. For a duration of 200 ns, we sampled the distributions of cations. First, we assessed if the simulations reached equilibrium by monitoring the observables in time (Figures S4–S6). Later, we analyzed how cations localize as the two DNA strands approach each other. To compare this behavior with isolated DNA pairs, we repeated the same analysis in the absence of lipids. Figure 4 illustrates the distributions of cations projected onto the axis orthogonal to the long DNA axis. The figures depict two- and one-dimensional concentration profiles along the interhelical distance. We present the cation occupancies normalized with respect to the bulk density. In the top view (Figure 4a–d), we show the DNA–lipid system where the lipid boundary is depicted by lines colored in gray. The black lines in one-dimensional plots represent the distribution of phosphate backbones on the DNA, while the green lines represent the average distribution of Mg^2+^ ions.

Interestingly, we observed discrete Mg^2+^ binding events that exhibited weak dependence on the positions of the DNAs. Notably, the cation concentration between the bilayers ranged from 1 to 2 M, despite the bulk concentration of Mg^2+^ ions being 50 mM. The cations are primarily localized at the membrane surface, with some also present at the interface. This localization at the interface became more prominent as the two DNA strands approached each other (Figure 4a–d).

In contrast to the lipid–DNA system, DNA pairs in aqueous media (Figure 4e–h) experienced a relatively diffusive counterion cloud, in sharp contrast to the discrete cation binding observed on the lipid bilayers. Here, Mg^2+^ ions tend to localize around the helices, and as the DNA pairs approach each other, cations accumulate in the depletion zone, resulting in high cation density at the interface.

Comparing the two cation distributions, we observed distinct differences between the DNA-in-lipid and the DNA-in-solution systems. The DNA-in-lipid system exhibited discrete and localized binding of cations, while the DNA in aqueous solution displayed diffusive cation dynamics. The discrete binding of Mg^2+^ cations in the presence of lipid-bound DNA supports earlier findings that the reduced dielectric permittivity of the medium induced by the membrane strengthens electrostatic interactions.^38,47^ To test this, we calculated the dielectric constants. As shown in Table S2, the lipid system created a low dielectric permittivity, ϵ_r_ = 40.1, in sharp contrast to the DNA in solution (ϵ_r_ = 92.2). Thus, the presence of the membrane resulted in reduced dielectric screening by the solvent, contributing to the enhanced attraction between oppositely charged species in the confined environment. Interestingly, the concentration of Mg^2+^ ions around the DNAs was higher in the aqueous phase, suggesting that DNA in CL membranes releases cations, leading to a gain in translational entropy of cations when DNA pairs are sandwiched between the lipid bilayer. This result is also consistent with earlier studies.^35,43−45,52^

Experimental findings have indicated that the localization of Mg^2+^ cations situated between DNA pairs contributes to DNA attraction in the presence of lipids.^29^ To scrutinize this hypothesis, we computed the average densities of Mg^2+^ ions located between DNA pairs within both the DNA-in-lipid system and DNA in an aqueous solution (Figure 4i,j). Our molecular dynamics simulations revealed that Mg^2+^ ions exhibited localization at the interface in the DNA-in-lipid scenario, confirming the measurements. However, we also observed a similar behavior in the DNA-in-solution case. Thus, while the presence of Mg^2+^ cations between DNA pairs may indeed contribute to DNA attraction within a lipid environment, our findings suggest that this behavior is not exclusive to the DNA-in-lipid system and can also manifest in DNA within aqueous solutions.

### Indirect and Direct Binding Modulate Membrane–DNA Interactions

**Figure 5:**
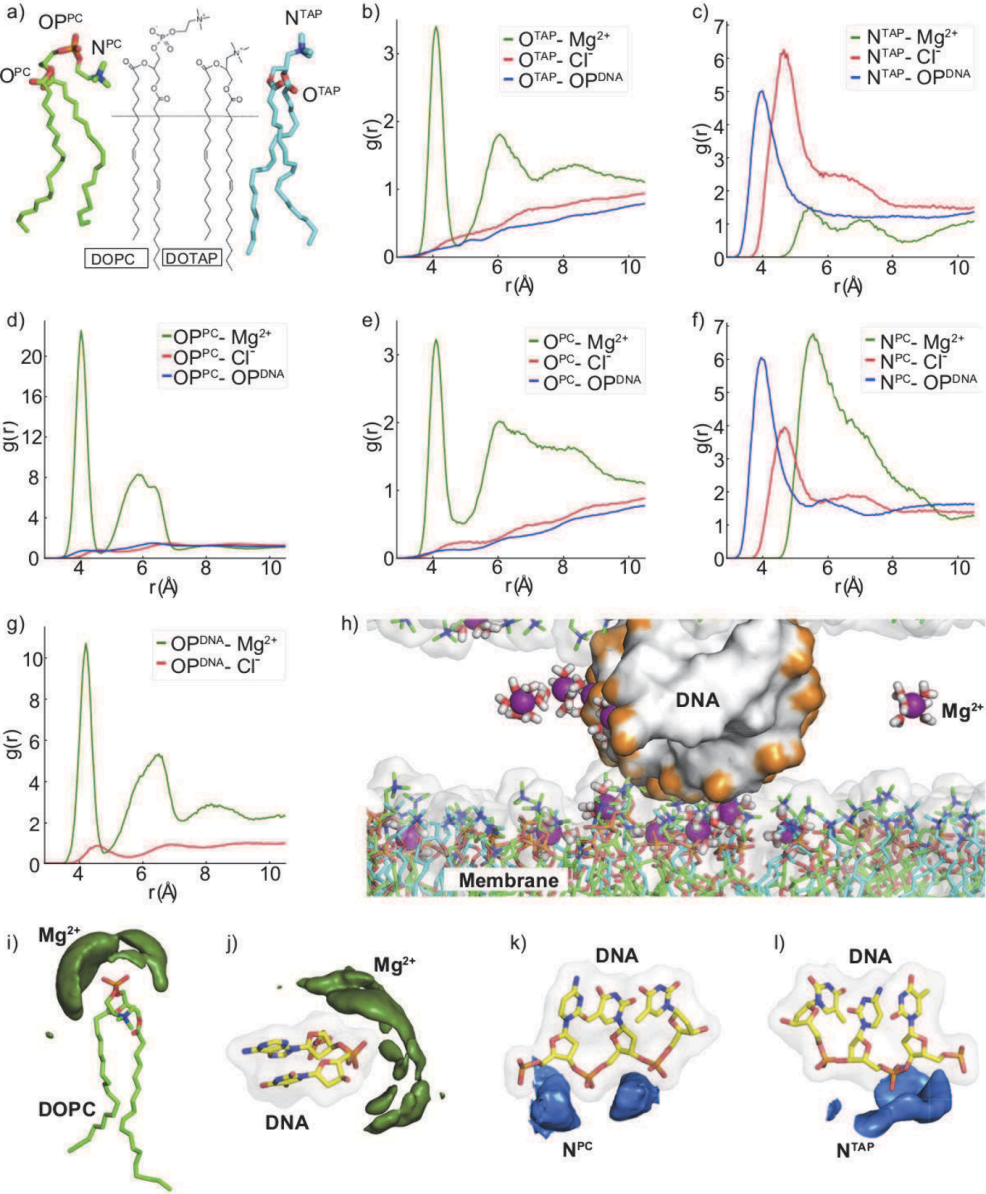
Radial distribution function (RDF) analysis of membrane–DNA and membrane–ion interactions. (a) Partitioning of the head groups of lipid molecules. (1) DOPC: ester group O^PC^, phosphate group OP^PC^, and amino group N^PC^. (2) DOTAP: O^TAP^ and N^TAP^. (b–g) RDF of each group with ions. (h) A snapshot from MD simulations illustrating the positioning of DNA and ions at the surface of the lipid membrane. Water molecules are removed for clarity. (i–l) Spatial distribution functions (SDFs) of the different species around their reference molecules were calculated from the MD simulations. Isosurfaces are shown in green for Mg^2+^ and in blue for lipid head groups (i.e., N^PC^ and N^TAP^).

We observe that the discrete binding of Mg^2+^ ions creates a unique distribution of cations on the surface of the membrane (Figure 4a–d). However, the question remains about the interactions between cations, lipids of the membrane, and the DNA. The partitioning of the cations and the charged groups in the membrane–DNA interface is not accessible by experiments and mean field theoretical approaches. To address this, we examined the specific interactions between the membrane head groups and the negatively charged DNA backbone. We divided the head groups of the membrane that face the DNA surface into the following sites: O^PC^, OP^PC^, and N^PC^ for DOPC and O^TAP^ and N^TAP^ for DOTAP (as shown in Figure 5a). Using radial distribution function (RDF) and spatial distribution function (SDF) analysis, we investigated how each site interacts with the ions and DNA backbone represented by the oxygens of the phosphate group (OP^DNA^).

Interestingly, we found that the positively charged DOTAP lipids did not exhibit strong binding to Mg^2+^ ions (Figure 5b,c). Negatively charged O^TAP^ on the other hand showed weak binding to the Mg^2+^ ions (Figure 5b) due to its buried position. O^TAP^ showed no binding to the DNA surface either, likely due to its like-charge nature. Unlike O^TAP^, the positively charged N^TAP^ head group formed a direct contact with the DNA backbone (Figure 5c), and Figure 5 depicts in detail the distribution of N^TAP^ in the vicinity of DNA. Interestingly, this binding is also accommodated by a notable Cl^–^ ion accumulation. Due to its positive charge, N^TAP^ did not show any binding to Mg^2+^ ions.

Unlike DOTAP, the negatively charged moieties of the head group in DOPC showed high correlations with Mg^2+^ ions (Figure 5d–f). The phosphate group (OP^PC^) exhibited the strongest affinity, and unsurprisingly, SDF analysis demonstrated the dense Mg^2+^ cloud around the OP^PC^ groups (Figure 5i). Interestingly, the OP^PC^–Mg^2+^ interactions were stronger than the OP^DNA^–Mg^2+^ interactions (Figure 5g,j), indicating that Mg^2+^ cations first bind to the membrane surface, creating additional positive sites and increasing the surface charge density of the cationic lipid. The increased charge density enhances the DNA–DNA ES interactions^35,47^ and contributes to the overall stabilization of the DNA–membrane complex. The oxygen of the ester group of DOPC, O^PC^, showed weaker interactions with both Mg^2+^ ions and DNA, likely due to its buried position and negative charge, respectively. Similar to N^TAP^, the positively charged nitro group of DOPC, N^PC^, directly contacted the DNA backbone (see Figure 5f,k), while its binding to Mg^2+^ ions was not direct; Cl ion binding was also weakly coordinated in comparison to N^TAP^.

Thus, our analysis leads to the conclusion that the interactions between the membrane and DNA occur through two distinct mechanisms (Figure S7). The first mechanism involves the direct binding of positively charged head group elements (N^TAP^, N^PC^) to the DNA phosphate oxygens (Figure S7a). This direct binding facilitates the attraction between the membrane and individual DNA pairs and potentially serves as a substrate for assembling negatively charged DNA duplexes onto the bilayer. We observed that there are ∼21–24 N^TAP^/N^PC^’s in direct contact with the negatively charged DNA surface throughout the condensation path constituting ≈75% of binding (Figure S7a). In addition to the positively charged moieties of head groups that show direct binding, the negatively charged groups of the membrane head groups participate in the DNA coordination through bridging (Figure S7b). This mode of binding is indirect and relies on the presence of Mg ions, and the number of Mg ions bridging the membrane and DNA backbone slightly increased as DNAs transitioned to the condensed state (Figure S7b). The two modes of interactions are depicted in a snapshot in Figure 5h and further detailed in Figure S7. These two mechanisms that we observe have long been established between divalent and negatively charged lipids.^66,67^

**Figure 6:**
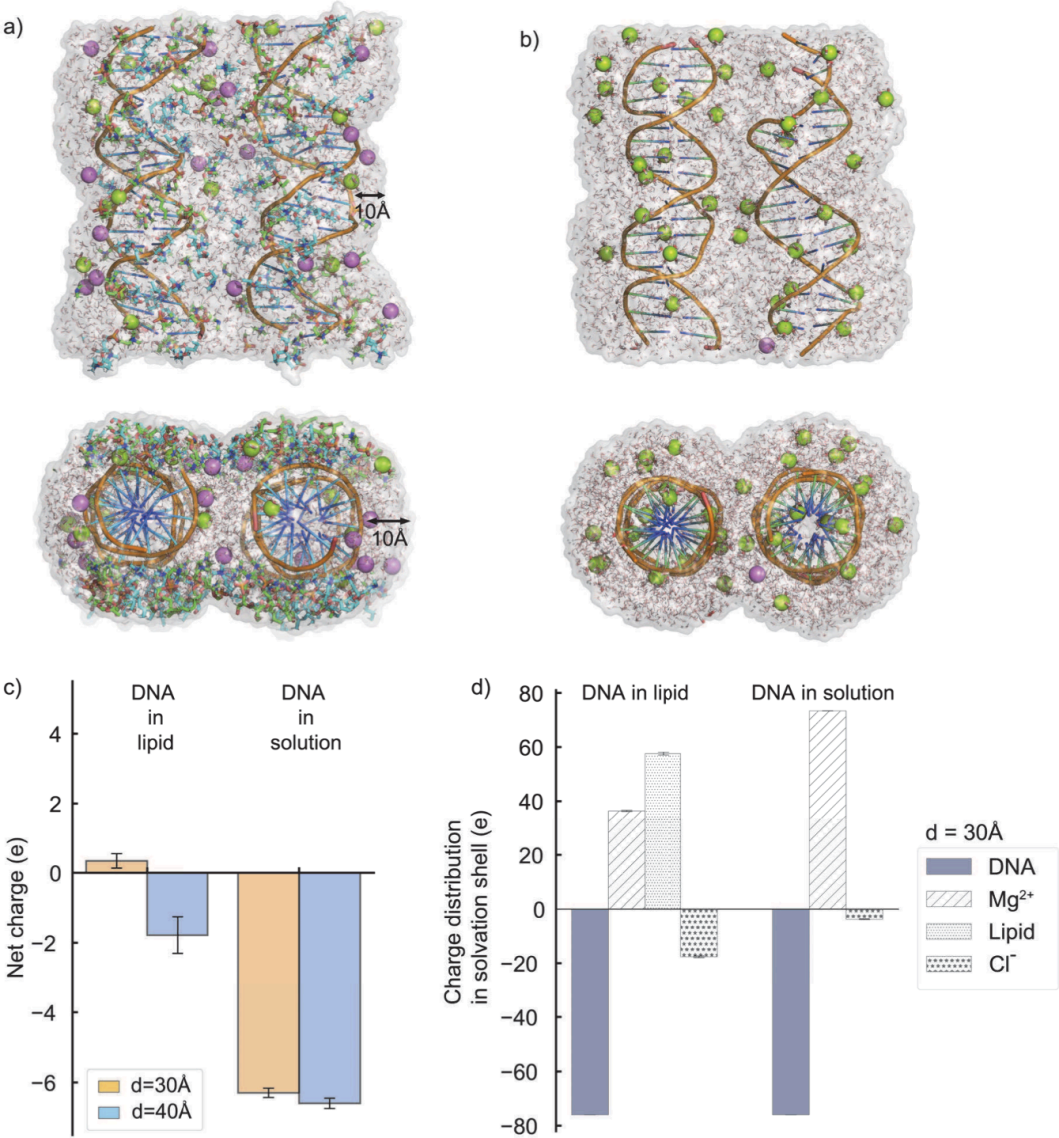
Solvation shell charge analysis. (a) Graphical representation of the DNA solvation shell selected for analysis, with a radius of 10.0 Å, for DNA in lipid. (b) Graphical representation of the DNA solvation shell selected for analysis, with a radius of 10.0 Å, for DNA in solution. (c) Comparison of the net charge in each solvation shell at the free state (*d* = 40 Å) and the condensed state (*d* = 30 Å). (d) Analysis of the charge distribution at the condensed state. A similar analysis for the free state can be found in Figure S3.

In summary, both the direct binding of positively charged head groups and the bridging of Mg^2+^ ions by negatively charged membrane groups play important roles in the interaction and stabilization of the DNA–membrane complex. To evaluate the contribution of all charge compensating mechanisms in reducing the overall electrostatic charges of DNA, we calculated the average net charge within a solvation shell of 10 Å around the DNA surface (Figure 6a,b). Although the choice of the 10 Å cutoff is somewhat arbitrary, it is larger than the Debye length (∼8 Å) in our salt condition, ensuring the validity of our analysis. Similar conclusions regarding charge partitioning can be drawn with different cutoff values. Through this exercise, we assessed whether the charge partitioning depends on the interhelical distance of the DNA. Furthermore, we compared the DNA charge environment within the lipid bilayer to that in aqueous solution to elucidate the unique role of membrane charge neutralization. Figure 6c shows our results.

Our analysis yielded an interesting finding. The net charge, denoted as *Q*_T_, within the solvation shell of DNAs wrapped by lipids showed a significant reduction compared to DNA in solution: *Q*_T_ ≈ −1.78 ± 0.52*e* versus *Q*_T_ ≈ −6.60 ± 0.15*e*, respectively. The interhelical distance of DNA pairs had a minor impact on the overall changes, with closer DNAs exhibiting slightly higher net charges. In the aqueous phase, the magnitude of the magnesium contribution is the most important factor in screening the DNA charge (*q*_Mg_ ≈ 73.4*e*). Remarkably, in the lowest energy state of the DNA–lipid complex (Figure 2a), the net charge approached neutrality: *Q*_T_ ≈ 0.34 ± 0.21*e*. Given the total bare DNA charge *q*_DNA_ = −76.0*e*, we found that Mg^2+^ ions contributed *q*_Mg_ ≈ 36.4*e*, which is only ≈48% of the negative charge of DNA, while the positive head groups of the membrane contributed ≈75% of DNA charges. The DNA with lipids and cations becomes positively charged, which induces an enhanced accumulation of Cl^–^ ions that balance the excess cation accumulation (*q*_Cl_ ≈ −17.6*e*) (Figure 6d). Additionally, ref (29) reported 0.63 Co^2+^ ion per base pair (bp); however, such measurements are not possible for Mg ions. Despite both Co^2+^ and Mg^2+^ having the same valence, their charge densities differ. Specifically, Co^2+^ induces condensation at around 24 mM while Mg^2+^ induces condensation only after reaching 48 mM, indicating that Mg is a weaker divalent ion. Consequently, the overall impact on Mg screening is anticipated to be less than 0.63 ion/bp. To verify this, we computed the Mg ion coordination using the radial distribution function (RDF). Our calculations yielded a value of approximately 0.46 Mg^2+^ ion/bp. This result closely aligns with our expectations based on the experimental trends in divalent charge density. Moreover, we assessed the impact of ion–ion correlations among ions confined between the two DNA strands in the condensed state using the equation Γ = *l*_B_/*d*,^68^ where *d* represents the average separation between the ions of interest, and *l*_B_ = *q*^2^/ϵ_r_*k*_B_*T* denotes the Bjerrum length. At this length, the electrostatic energy between two ions of charge *q* in a medium with relative permittivity ϵ_r_ equals *k*_B_*T*. Unsurperisingly, our calculations have demonstrated that the Mg^2+^–Mg^2+^ ion correlations in lipid environments are significantly stronger than those observed in solution systems ( ∼ 1.87 in the DNA-in-lipid system,  ∼ 0.84 in the DNA-in-solution system), thereby enhancing the DNA–DNA attraction. The correlations between Mg^2+^ and Cl^–^ ions are considerably weaker in both cases ( ∼ 0.63 in the DNA-in-lipid system,  ∼ 0.28 in the DNA-in-solution system), stemming from the low concentration of Cl^–^ ions in the interstitial gap between DNA strands. This highlights that the distinct ionic interactions affect the structural dynamics and stability of DNA within different media.

Overall, simulations demonstrate that the combination of direct binding of head groups and bridging Mg^2+^ ions leads to a significant reduction in the net charge within the solvation shell of the DNA–membrane complex. Mg^2+^ ions play a prominent role in charge compensation, while the positive head groups of the membrane contribute more significantly to neutralizing the negative charge of DNA in the case of the membrane–DNA complex. Furthermore, there is a dramatic reduction of Mg coordination upon binding of the DNA to the lipid membrane, supporting earlier observations. The release of Mg^2+^ cations into the solution provides additional support to stabilize the DNA–membrane complex. While the bridging and release of Mg cations contribute to the stabilization of the DNA–membrane complex, the precise mechanism through which confined divalent cations induce attraction between DNA molecules remains uncertain.

### A Statistical Thermodynamics Model to Explain DNA Condensation in Cationic Lipid Bilayers

**Figure 7:**
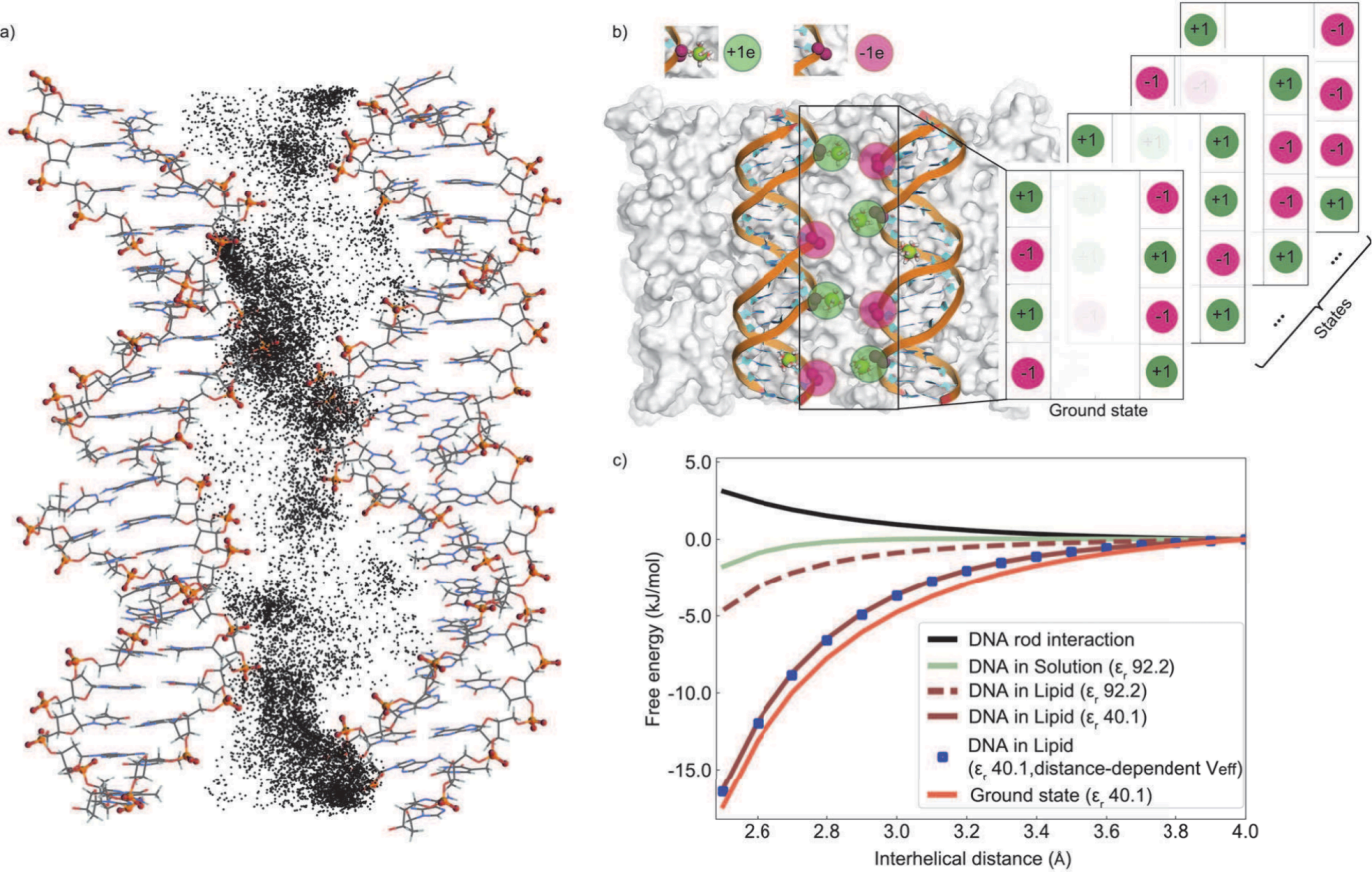
Statistical thermodynamics model of attraction induced by cation fluctuations. (a) The trajectory of a monitored Mg^2+^ ion hopping between the phosphate groups. (b) Mg^2+^ bound to phosphate binding sites (green circles) creates a +1*e* charge, while unoccupied phosphate groups (purple circles) possess a −1*e* charge. The model considers all possible binding states. (c) The resulting free energy profiles, taking into account charge-fluctuation-induced attraction and DNA–DNA repulsion, are plotted as a function of interspacing for different scenarios.

To understand the behavior of trapped cations between DNA pairs, we monitored each cation in the simulation. We observed that counterions trapped between bilayers move along the phosphate groups between DNA strands like a ping-pong ball moves between the two sides of the tennis table. This behavior is exemplified by the trajectory of a typical Mg^2+^ ion as illustrated in Figure 7a. Can cations of such confined between the two DNA strands induce attraction? To answer this question, we propose the following simple model shown in Figure 7b.

The magnesium ions move between the phosphate groups, creating charge fluctuations between the two DNA strands. When the distance between two adjacent DNA strands is short enough, with *x* < κ^–1^, where κ^–1^ is the Debye length, the counterions confined at the interface of the two parallel DNA strands contribute most to the electrostatic interaction. The counterions confined between DNA strands are expected to move along the phosphate groups where the electrostatic interactions are the highest. We assume each phosphate group possesses a charge of −1*e*. To simplify the analysis, we consider only the phosphate groups at the interface, as shown in Figure 7b, where *b* represents the average distance between such groups along the same DNA strand of length, *L.* Following ref (69), the electrostatic interaction resulting from ion correlations between the two DNA strands can then be estimated by considering all possible cation binding configurations, as described by the following energy term:
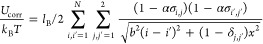
4where σ_*ij*_ is the occupation variable, with *i* = 1, 2, ..., *N* representing the sites in the first DNA, while *i*′ = 1, 2, ..., *N* is for the second DNA. *N* ≈ *L*/*b* is the total number of interface phosphate groups involved. The variable *j* = 1, 2 distinguishes whether the site is occupied or not. α is the valence of counterions, which is +2 in the case of Mg^2+^. Following this rationale, σ_*ij*_ = 1 indicates that the binding site is occupied by Mg^2+^ ions, resulting in a net charge of +1*e*, while σ_*ij*_ = 0 indicates an unoccupied site with a charge of −1*e*.

In addition to the correlation term, the partially screened DNA molecules interact with each other electrostatically. Here, we assume DNAs are homogeneously charged rods. From the Poisson–Boltzmann equation, the reduced potential of a charged rod in a salt solution has the form^70^
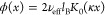
5where  is the Bjerrum length, with ϵ_0_ and ϵ_r_ the electric permittivity in vacuum and the relative permittivity in water or in lipid that is directly calculated from the explicit simulations, respectively, *k*_B_*T* is the thermal energy, ν_eff_ is the effective charge after the cation condensation happens, *K*_0_ denotes the zero order Bessel function, and κ is the inverse of the Debye length, defined as , where *I*_*M*_ is the ionic strength expressed in molar. Based on this, the mean-field theory of the total electrostatic interaction energy of two rods separated by a distance *x* is given by
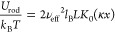
6Note that this interaction is always repulsive.

Based on the Hamiltonian, which is a function of the interspacing *x*, i.e., *H*(*x*) = *U*_rod_(*x*) + *U*_corr_(*x*), we write the grand canonical free energy with respect to *x*:
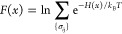
7

We evaluate eq 7 numerically once we set *b* ≈ 1.75 nm and *L* = 6.8 nm obtained from the simulations. Also, from the simulations we compute ν_eff_ at the free state (i.e., *d* = 40 Å) for lipid-bound DNA and DNA in solution systems, ν_eff_ ≈ – 1*e*/20 bp and ν_eff_ ≈ – 3.5*e*/20 bp, respectively, and ν_eff_ can be assumed to vary linearly with changes in interhelical spacing *d*. The free energy change from the statistical model is shown in Figure 7c. Consistent with Figure 2a,b, our free energy profiles show attraction in the lipid–DNA system while remaining repulsive in the case of DNA in solution. It is important to highlight that the free energy of the bilayer system closely approaches ground state energy, where the sites exhibit an alternating binding pattern (Figure 7b). This observation implies that in the presence of lipids thermal energy between the bilayers does not significantly influence the outcome; instead, the electrostatic energy plays a major role in governing the processes. It is noteworthy that this conclusion contrasts with the situation observed in divalent ion induced like-charge attraction that takes place in solution.^61,71^ It is important to mention that our model only looks at the electrostatic interactions while MD simulations consider many factors such as dispersion forces, lipid dynamics, depletion, hydration, hydrodynamics, and many more, thereby only leading to modest quantitative agreement in free energy between the simulations and model predictions. Incorporating the factors noted above into the model could achieve more quantitative agreement with the simulation, but that is beyond the current scope of our study.

## Conclusion

Our objective was to investigate the factors that contribute to DNA condensation in divalent cations within cationic lipid bilayers. To achieve this, we utilized all-atom detailed molecular dynamics simulations, combined with extensive conformational sampling, to explore the free energy landscape of DNA–DNA interactions. Through our analysis, we were able to identify the key elements responsible for the attraction of DNA between lipid bilayers. Our findings indicate that the condensation of DNA pairs is primarily driven by electrostatic interactions between lipids and DNA. Specifically, we demonstrated that cationic lipids play a significant role in shielding the DNA, thereby reducing the overall repulsive forces. Moreover, the presence of divalent cations confined between adjacent DNA pairs creates charge fluctuations, generating a driving force that leads to spontaneous condensation between the negatively charged DNA duplexes. To further validate our observations, we conducted simulations where we removed the lipid bilayers surrounding the DNA. This resulted in poor charge screening. Despite the favorable charge fluctuations, the repulsive forces between the DNA pairs overcome the charge fluctuations, leading to repulsion as observed by experiments. Our study highlights the importance of charge screening accomplished by lipid head groups as the major contributor to lipid-induced DNA condensation.

We have observed that divalent metal ions that interact with the negatively charged phosphate groups of the lipid head groups and DNA promote the binding of DNA to the membrane. This observation aligns with earlier reports in other studies.^49,52^ Our simulations have also revealed an intriguing phenomenon concerning the distribution of cations sandwiched between lipid bilayers. Instead of a mobile cation atmosphere that uniformly surrounds the DNA as in an aqueous environment, we observed the formation of discrete binding sites in the presence of lipid bilayers. This discrete binding of cations is likely a consequence of the reduced local dielectric permittivity at the interface of the membrane surface, as suggested in previous studies^38,47^ and in our simulations (Table S2). The binding mechanisms noted above have long been established for many types of charged biopolymers and lipid membranes. For instance, divalent ions bridge the interaction of DNA with zwitterionic lipids and mediate the DNA assembly.^72,73^ Divalent cation bridge binding also facilitates the binding of amyloid-like peptides to a lipid bilayer^74^ and induces the cluster formation of polyphosphoinositides on the membrane.^75^ It is also important to note that divalent cations, such as Ca^2+^ and Mg^2+^, can modulate the membrane configurations by inducing different hydration structures of the membrane, due in part to their different binding sites on lipid bilayers.^76,77^ This factor merits inclusion in our model as part of future investigations.

In the presence of lipids, these discrete cation binding sites act as bridges, connecting the DNA strands to the surface of the membrane.^49,78^ The negatively charged lipids, decorated with divalent cations, coordinate with the phosphate backbone of DNA to facilitate binding. Moreover, we observe that the positively charged lipid head groups expel counterions bound to DNA to the solution, increasing the translational entropy of the counterions, a mechanism proposed in other theoretical studies.^38,47,79^ The increased mobility of bound cations facilitates the binding of DNA molecules to CL membranes, forming a lamellar CL–DNA complex. While all these factors are supportive, using a statistical thermodynamics model that emphasizes the role of counterion dynamics, we show that it is the charge correlations that drive the already screened DNA pairs to the condensed state.

Although we provide an atomically detailed description of DNA condensation inside cationic bilayers, we note that other lipid compositions, such as DMPC/DMTAP,^37^ DOPE/DOTAP,^31^ and even anionic lipids like DOPC/DOPG, have been observed to form condensates, leading to lamellar or hexagonal DNA–lipid complexes.^30^ Further research will aim to explore their unique mechanisms. In addition, the exact mechanisms by which ion type and concentration influence DNA like-charge attraction remain unclear and warrant further investigation.

In summary, our simulation study sheds light on the intricate processes underlying DNA condensation in lipid bilayers, underscoring the significance of electrostatic interactions modulated by cationic lipids and divalent cations. We have identified the dynamics of divalent counterions and charged lipid head groups as key factors facilitating this condensation. Lipids exhibit the ability to self-assemble into diverse structures in aqueous solutions,^80−82^ with the planar bilayer being particularly relevant in biological systems. The phenomenon of macroion adsorption onto oppositely charged lipid membranes is not only prevalent in living cells but also finds applications in various technological and pharmaceutical contexts. Therefore, gaining a deeper understanding of the underlying principles of this phenomenon has the potential to advance the field.

## Supporting Information Available

Molecular simulation setups for the two DNA systems under study, brute force MD and well-tempered metadynamics simulation details, details of computing dielectric constant from simulations, details of cation analysis for the DNA-in-lipid system (PDF)
