# Supplementary material for: Mechanism of Cationic Lipid Induced DNA Condensation: Lipid–DNA Coordination and Divalent Cation Charge Fluctuations
Source: Biomacromolecules. 2024 Jul 16;25(8):4819–30. doi: 10.1021/acs.biomac.4c00192 (PMC11323003; doi:10.1021/acs.biomac.4c00192)
Supplement: Supplementary file 1 — bm4c00192_si_001.pdf [file bm4c00192_si_001.pdf]

Supporting information for Mechanism of Cationic Lipid-Induced DNA Condensation: Lipid-DNA coordination and divalent cation charge fluctuations

Weiwei He[†,‡] and Serdal Kirmizialtin[*,†,‡]

†*Chemistry Program, Science Division, New York University Abu Dhabi*

‡*Department of Chemistry, New York University*

E-mail: serdal@nyu.edu

Simulation setup of DNA in solution

We study GCA TCT GGGC TATA AAA GGG and its complementing sequence. We generated the structure of the duplex by Nucleic Acid Builder (NAB)[1] and placed the two duplexes of the same sequence in the simulation box with initial dimensions of 11.8x11.8x6.8 nm^3. We set the long axis of DNA constructs to align parallel to box's z-axis so that the DNAs could be extended to infinite length due to periodic boundary conditions (PBC). 5000-step steepest descent energy minimization was employed to refine the initial structures. The box was then solvated with water and ions to satisfy charge neutralization, resulting in free salt condition of ~50mM $MgCl_2$. For all simulations, the GROMACS 2018.5 package [2] was used amber99sb_parmbsc0 force field for nucleic acids, NBFIX[3] for both mono- and divalent ions (i.e. Cl^- and hexa-hydrated Mg^{2+}), and TIP3P[4] for water. The electrostatic interaction was treated using the particle mesh Ewald method (PME)[5] where a grid spacing of 0.12 nm and an interpolation of order 4 were used, with a real space and van der Waals cutoff distance of both 1.1 nm. The equations of motion were solved using the Leapfrog scheme with a time step of 2 fs.

The solvated system was energy minimized with the steepest descent algorithm for 5000 steps to remove the bad contacts due to random replacement of water molecules and ions. The minimized structures were then equilibrated for volume and solvent. For that, we first performed 2.5-ns-long simulation in isothermal-isobaric (NPT) ensemble at 300K and 1 bar, ensured by Berendsen thermostat and Parrinello-Rahman barostat, respectively. During this step, all DNA heavy atoms were restrained by harmonic restraints with a stiffness constant of 1000 $kJ \cdot mol^{-1} \cdot nm^{-2}$ to their initial positions, while ions and water were left free to move. Subsequently, using the coordinates of the last snapshot of this NPT simulation and keeping the position restraints, we run for another 200 ns long MD simulation in canonical ensemble (NVT) to equilibrate the distributions of cations and water. This step ensures the counter ion atmosphere equilibriates around the DNA pairs. The last frame of this equilibration simulation was taken for the metadynamics simulations. Additional details of the simulation

setup can be found in Table S1.

Simulation setup of DNA in lipid membrane

Initial coordinates and topology for membrane systems were built using a lipid bilayer builder embedded in CHARMM-GUI.[6] To be consistent with experimental conditions, we used the pre-equilibrated lipid bilayer systems containing 122 DOPC and 82 DOTAP lipid molecules (mole fraction of DOPC Φ_{DOPC} $\sim$0.6[7]). The Amber force field was extended to include the topological information for the membrane, with parameters taken from the CHARMM-GUI interface.[8] Consequently, the simulation box of DNA in the lipid system had initial dimensions of 10.2x7.0x6.8 nm^3 and was then solvated with water. Before adding ions, the water molecules buried in the interior of the membrane, caused by random insertions during solvation, were carefully removed to avoid improper interactions.[9,10] Except for the extended topology, all other simulation parameters were similar to the settings in the simulations of the DNA in solution system (see Section **Simulation setup of DNA in solution**). Likewise, the solvated system was energy minimized and then equilibrated with heavy atoms of both DNA and membrane restrained in NPT and NVT ensemble, respectively. In addition, a two-group temperature coupling protocol was employed separating DNA+ions+water system, and membrane. The last snapshot of the NVT trajectory was used to start the metadynamics simulations. In metadynamics, the lipid head groups were restrained by soft harmonic restraints to maintain the bilayer structure. Additional simulation details can be found in Table S1.

Calculation of dielectric constant

To assess the reduced local dielectric permittivity at the interface of the membrane surface,,[11,12] we computed the static dielectric constant from the simulations using the Einstein-

Helfand method.[13,14]

$$\epsilon_r = 1 + \frac{\langle M^2 \rangle - \langle M \rangle^2}{3\epsilon_0 V k_B T}, \tag{1}$$

where variance $\langle M^2 \rangle - \langle M \rangle^2$ measures the dipole moment fluctuation, and the average dipole moment $\langle M \rangle$ was determined through the equation:

$$M = \sum_i q_i r_i \tag{2}$$

where r_i denotes the position of the i-th atom and q_i represents its charge. The dielectric constant was subsequently calculated employing the GROMACS dipoles utility, with epsilon of the reaction field set to default ($\epsilon_{RF} = \infty$).

Table S1

Simulation system	DNA in lipid [a]	DNA in solution [b]
Salt condition	24 $Mg(H_2O)_6{}^{2+}$<br>54 Cl^-<br>5581 H_2O	65 $Mg(H_2O)_6{}^{2+}$<br>54 Cl^-<br>30568 H_2O

[a] 10.2x7.0x6.8 nm^3, [b] 11.8x11.8x6.8 nm^3.

Table S2: Dielectric constant ϵ_r for the studied systems.

System	$C_{MgCl_2}(mol/L)$	ϵ_r
DNA in solution	0.05	92.2
DNA in lipid		40.1

Supplemental figures

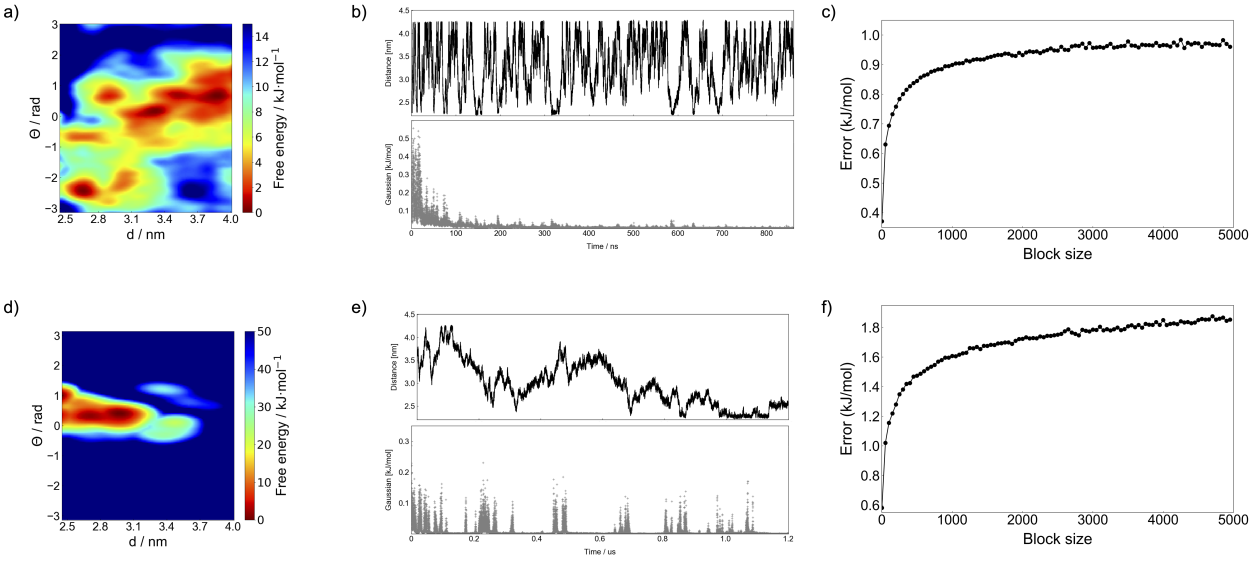

Figure S1: Free energy calculations for DNA in lipid versus DNA in solution. a-c) DNA in solution. a) free energy profile projected onto d and θ; b) time evolution of CV d and height of deposited Gaussian potential during metydynamics; c) Block analysis of convergence. d-f) The same analysis, this time for DNA in lipid.

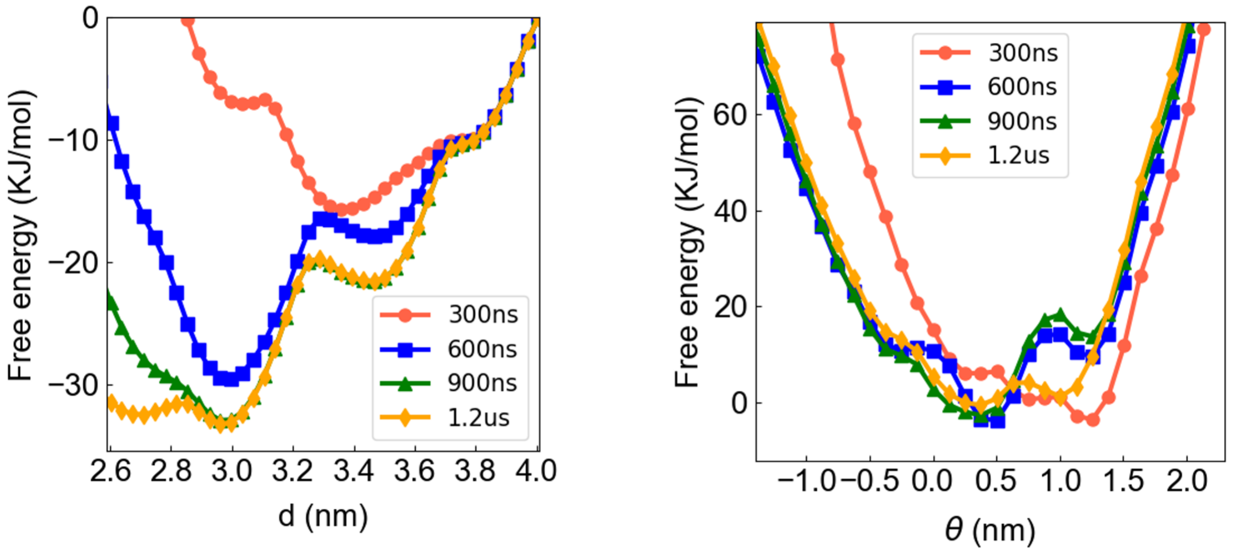

Figure S2: Free energy profiles of DNA in lipid from different metadynamics simulation time, as a function of the inter-spacing d and azimuthal angle θ.

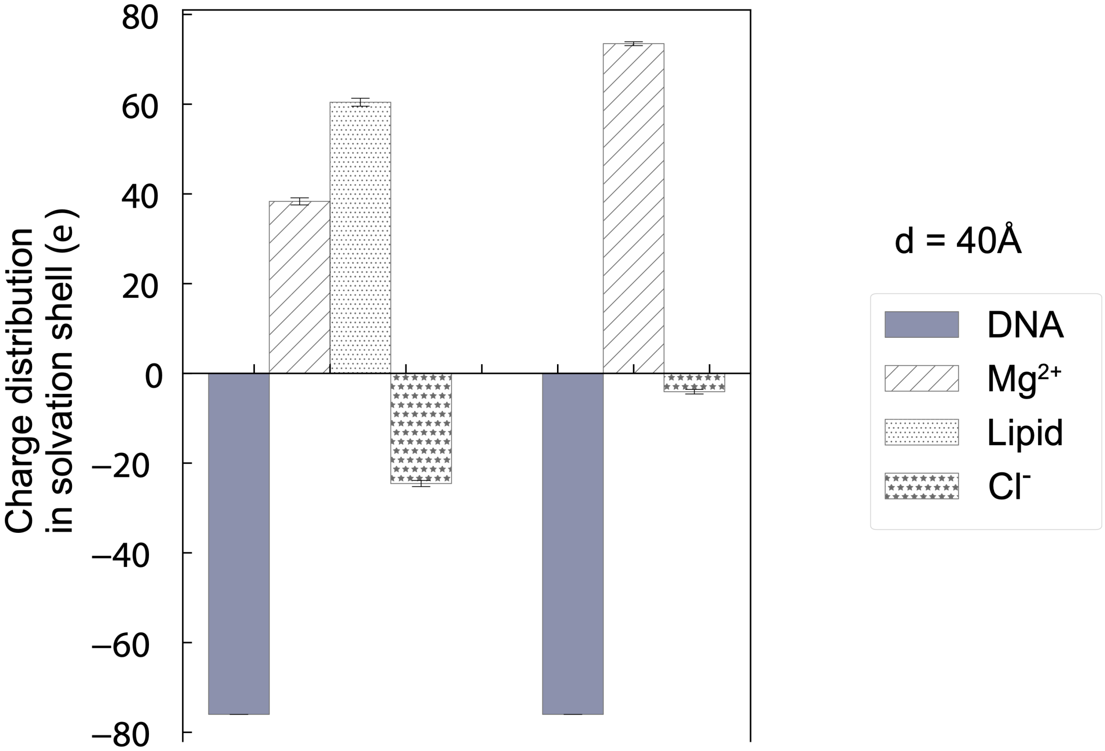

Figure S3: The charge distribution in solvation shell for DNA in lipid at d=40Å.

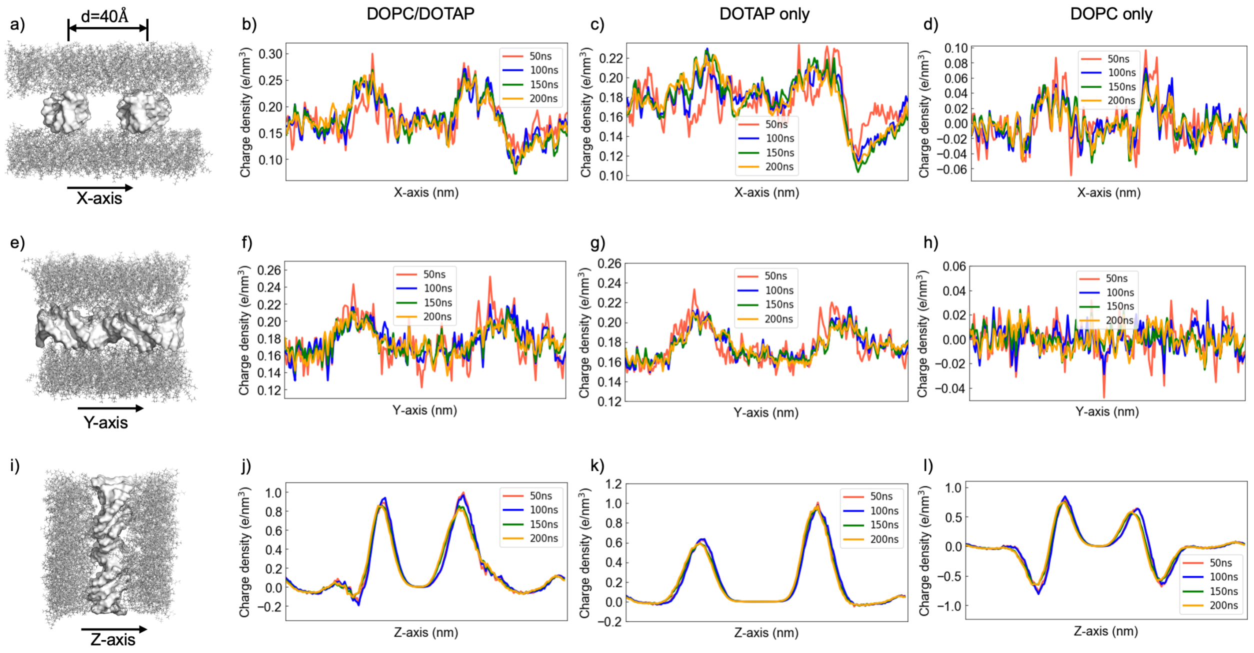

Figure S4: The charge density distribution of lipid bilayer along X-, Y- and Z-axis, at d=40Å, and its changes as a function of simulation time.

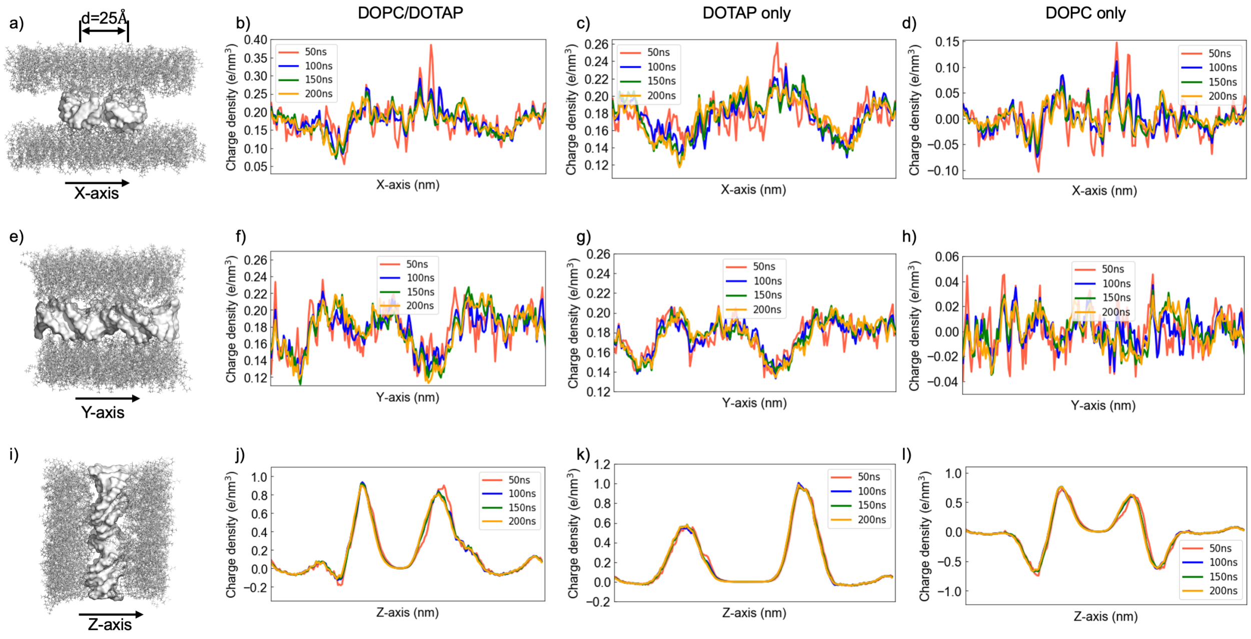

Figure S5: The charge density distribution of lipid bilayer along X-, Y- and Z-axis, at d=25Å, and its changes as a function of simulation time.

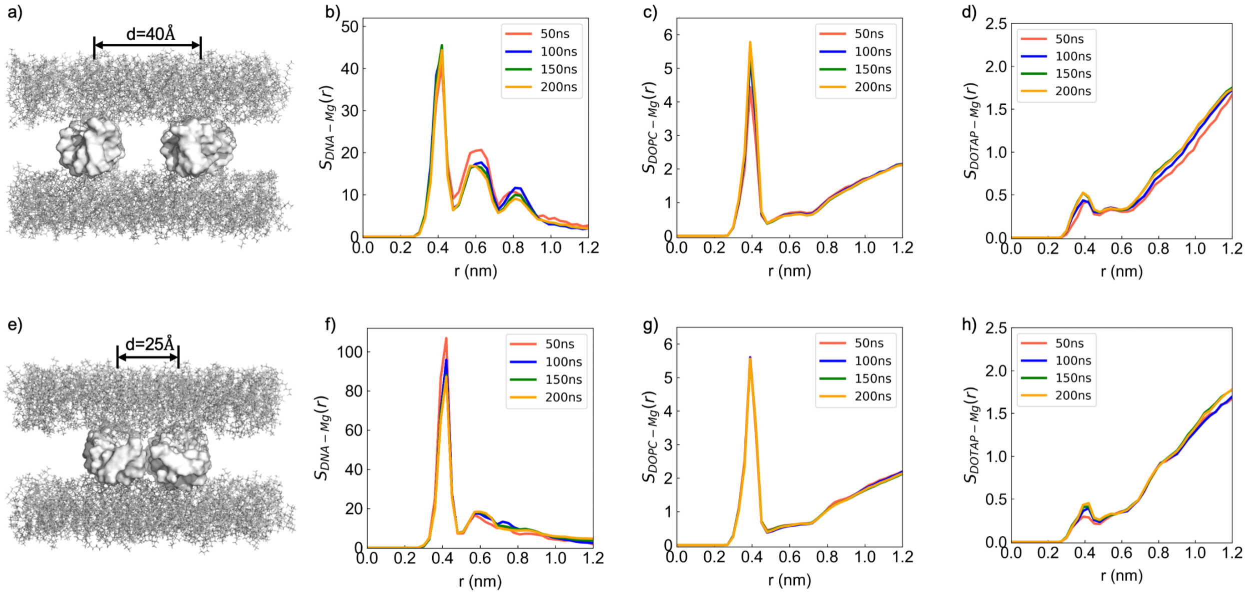

Figure S6: The surface radial distribution function, $S(r)$, of Mg^{2+} cations around the DNA duplex and lipid groups, and its changes as a function of simulation time

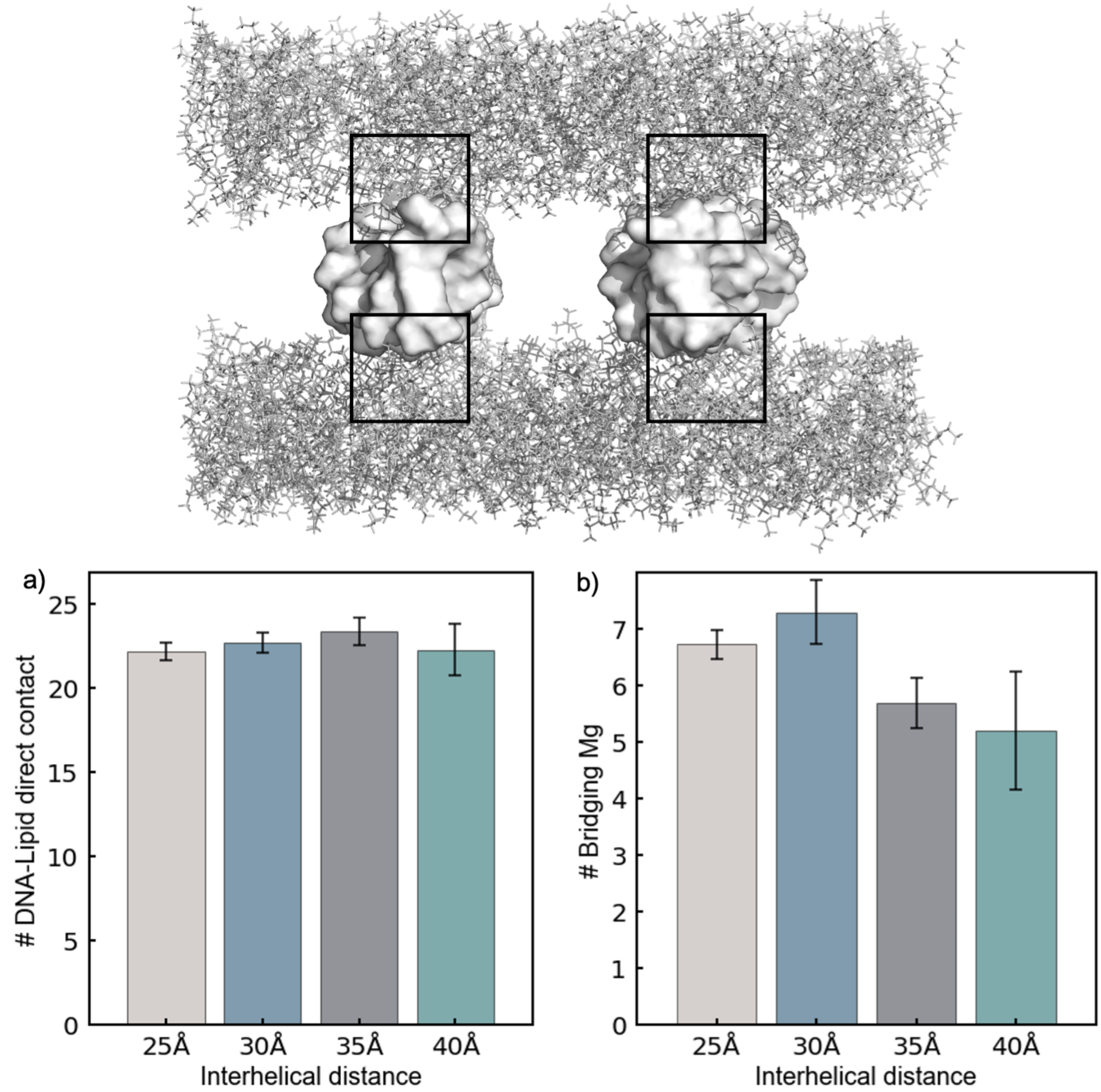

Figure S7: The number of direct DNA-membrane binding (defined for $d_{OP^{DNA}-N^{PC/TAP}} \leq 4.0$Å, corresponding to the location of the first peak in the RDF as shown in Fig. 5c,f.) and the number of Mg^{2+} ions bridging membrane and DNA surfaces, as a function of inter-helical separation.

References

(1) Case, D. A.; Cheatham III, T. E.; Darden, T.; Gohlke, H.; Luo, R.; Merz Jr, K. M.; Onufriev, A.; Simmerling, C.; Wang, B.; Woods, R. J. The Amber biomolecular simulation programs. *J. Comput. Chem.* **2005**, *26*, 1668–1688.

(2) Hess, B.; Kutzner, C.; Van Der Spoel, D.; Lindahl, E. GROMACS 4: algorithms for highly efficient, load-balanced, and scalable molecular simulation. *J. Chem. Theory Comput.* **2008**, *4*, 435–447.

(3) Yoo, J.; Aksimentiev, A. Improved parametrization of Li+, Na+, K+, and Mg2+ ions for all-atom molecular dynamics simulations of nucleic acid systems. *J. Phys. Chem. Lett.* **2012**, *3*, 45–50.

(4) Jorgensen, W. L.; Chandrasekhar, J.; Madura, J. D.; Impey, R. W.; Klein, M. L. Comparison of simple potential functions for simulating liquid water. *J. Chem. Phys.* **1983**, *79*, 926–935.

(5) Darden, T.; York, D.; Pedersen, L. Particle mesh Ewald: An N log (N) method for Ewald sums in large systems. *J. Chem. Phys.* **1993**, *98*, 10089–10092.

(6) Jo, S.; Kim, T.; Iyer, V. G.; Im, W. CHARMM-GUI: a web-based graphical user interface for CHARMM. *J. Comput. Chem.* **2008**, *29*, 1859–1865.

(7) Koltover, I.; Wagner, K.; Safinya, C. R. DNA condensation in two dimensions. *Proc. Natl. Acad. Sci. U.S.A.* **2000**, *97*, 14046–14051.

(8) Lee, J.; Cheng, X.; Jo, S.; MacKerell, A. D.; Klauda, J. B.; Im, W. CHARMM-GUI input generator for NAMD, GROMACS, AMBER, OpenMM, and CHARMM/OpenMM simulations using the CHARMM36 additive force field. *Biophys. J.* **2016**, *110*, 641a.

(9) Comer, J. R.; Wells, D. B.; Aksimentiev, A. Modeling nanopores for sequencing DNA. *DNA Nanotechnology: Methods and Protocols* **2011**, 317–358.

(10) Aksimentiev, A.; Sotomayor, M.; Wells, D. Membrane Proteins Tutorial. *University of Illinois at Urbana-Champaign* **2012**,

(11) Cherstvy, A. Electrostatics of DNA complexes with cationic lipid membranes. *J. Phys. Chem. B* **2007**, *111*, 7914–7927.

(12) Cherstvy, A. Electrostatic interactions in biological DNA-related systems. *Phys. Chem. Chem. Phys.* **2011**, *13*, 9942–9968.

(13) Schröder, C.; Haberler, M.; Steinhauser, O. On the computation and contribution of conductivity in molecular ionic liquids. *J. Chem. Phys.* **2008**, *128*, 134501.

(14) Neumann, M. Dipole moment fluctuation formulas in computer simulations of polar systems. *Mol. Phys.* **1983**, *50*, 841–858.
